# Functional roles of non-coding Y RNAs

**DOI:** 10.1016/j.biocel.2015.07.003

**Published:** 2015-09

**Authors:** Madzia P. Kowalski, Torsten Krude

**Affiliations:** Department of Zoology, University of Cambridge, Downing Street, Cambridge CB2 3EJ, United Kingdom

**Keywords:** sbRNA, stem-bulge RNA, RNP, ribonucleoprotein, PTB, polypyrimidine tract-binding protein, ZBP1, zipcode binding protein 1, Yrl RNA, Y RNA-like RNA, snRNA, small nuclear RNA, UV, ultraviolet, PNPase, polynucleotide phosphorylase, RYPER, Ro60/Y RNA/PNPase Exoribonuclease RNP, NMR, nuclear magnetic resonance, CD, circular dichroism, RNAi, RNA interference, MO, antisense morpholino oligonucleotide, MBT, mid-blastula transition, ORC, origin recognition complex, RoBPI, RoRNP binding protein I, YsRNAs, Y RNA-derived small RNAs, Non-coding RNA, Y RNA, DNA replication, RNA stability, RNA domains

## Abstract

Non-coding RNAs are involved in a multitude of cellular processes but the biochemical function of many small non-coding RNAs remains unclear. The family of small non-coding Y RNAs is conserved in vertebrates and related RNAs are present in some prokaryotic species. Y RNAs are also homologous to the newly identified family of non-coding stem-bulge RNAs (sbRNAs) in nematodes, for which potential physiological functions are only now emerging. Y RNAs are essential for the initiation of chromosomal DNA replication in vertebrates and, when bound to the Ro60 protein, they are involved in RNA stability and cellular responses to stress in several eukaryotic and prokaryotic species. Additionally, short fragments of Y RNAs have recently been identified as abundant components in the blood and tissues of humans and other mammals, with potential diagnostic value. While the number of functional roles of Y RNAs is growing, it is becoming increasingly clear that the conserved structural domains of Y RNAs are essential for distinct cellular functions. Here, we review the biochemical functions associated with these structural RNA domains, as well as the functional conservation of Y RNAs in different species. The existing biochemical and structural evidence supports a domain model for these small non-coding RNAs that has direct implications for the modular evolution of functional non-coding RNAs.

## Graphical abstract

**Figure:**
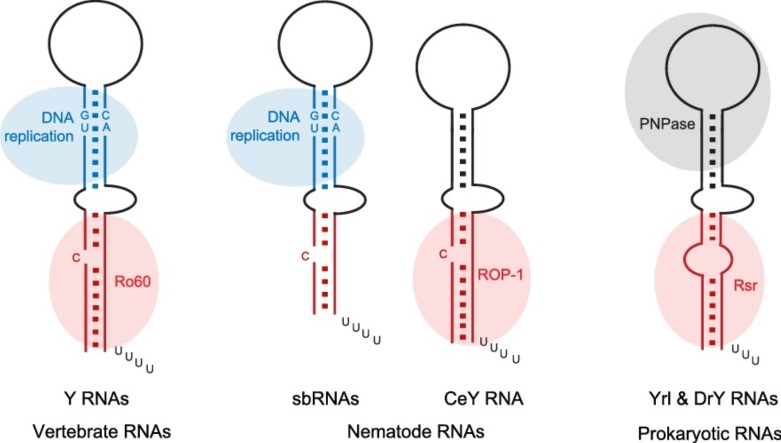

## 1 Introduction and historical overview

Small non-coding Y RNAs were first discovered in 1981 as components of ribonucleoproteins (RNPs) complexed with Ro60 and La proteins, autoantigens which are targets of the immune system in patients suffering from the autoimmune diseases systemic lupus erythematosus (SLE) and Sjögren's syndrome (Hendrick et al., 1981, Lerner et al., 1981). These non-coding RNAs were initially found in the cytoplasm of mammalian cells (human, mouse and monkey) and were therefore given the prefix ‘Y’, for cytoplasmic Y RNAs, to distinguish them from nuclear U RNAs (Lerner et al., 1981).

**Fig. 1:**
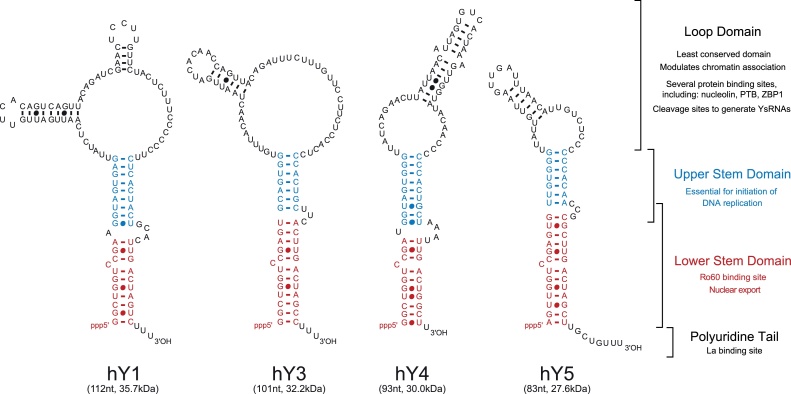
The non-coding human Y RNAs. The nucleotide sequences and secondary structures of hY RNAs are derived from sequence alignment and enzymatic and chemical probing (Teunissen et al., 2000, van Gelder et al., 1994). The conserved structural RNA domains and their associated functions are highlighted for each hY RNA. The size in nucleotides (nt) and molecular weight (kDa) of each RNA is indicated. See main text for references.

There are four non-coding Y RNAs in humans (hY1, hY3, hY4 and hY5 RNA; an hY2 RNA was also originally described, but was later removed from the list as it was found to be a degradation product of hY1 RNA). Y RNAs are present in all vertebrate species investigated so far, with between one and four different genes per species reflecting gene loss and duplication events during vertebrate evolution (Mosig et al., 2007, Perreault et al., 2007). In humans, the four Y RNA genes are clustered together at a single chromosomal locus on chromosome 7q36 (Maraia et al., 1994, Maraia et al., 1996). A similar syntenic arrangement of Y RNA genes has been described in other vertebrates (Farris et al., 1996, O’Brien et al., 1993). Individual Y RNA genes are transcribed by RNA polymerase III from distinct promoters (Hendrick et al., 1981, Wolin and Steitz, 1983). Y RNAs are relatively small at 100 ± 20 nucleotides in size, and fold into characteristic stem-loop secondary structures (Fig. 1). Chemical and enzymatic structure probing experiments have revealed that the 5′ and 3′ RNA ends hybridise to form predominantly double-stranded upper and lower stem domains with an internal loop (Teunissen et al., 2000, van Gelder et al., 1994). The nucleotide sequences of the lower and upper stems are highly conserved, whereas the sequences – and hence predicted structures – of the internal loop vary greatly between individual Y RNAs.

The existence of Y RNAs is not restricted to vertebrates. The family of small non-coding stem-bulge RNAs (sbRNAs) in nematodes has recently been shown to be homologous in structure and function to vertebrate Y RNAs (Boria et al., 2010, Kowalski et al., 2015). Small non-coding RNAs bearing similarities to vertebrate Y RNAs or nematode sbRNAs have also been reported in other eukaryotes, including the insects *Anopheles gambiae* (Perreault et al., 2007) and *Bombyx mori* (Duarte et al., 2015), and the lancet *Branchiostoma floridae* (Mosig et al., 2007). Similar RNAs have also been described in some prokaryotes, including *Deinococcus radiodurans* (Chen et al., 2000), *Salmonella enterica* serovar Typhimurium (Chen et al., 2013), *Mycobacterium smegmatis* and possibly many more (Chen et al., 2014). These bacterial RNAs are highly divergent from, and not homologous with, the four vertebrate Y RNA clades (Perreault et al., 2007).

**Fig. 2:**
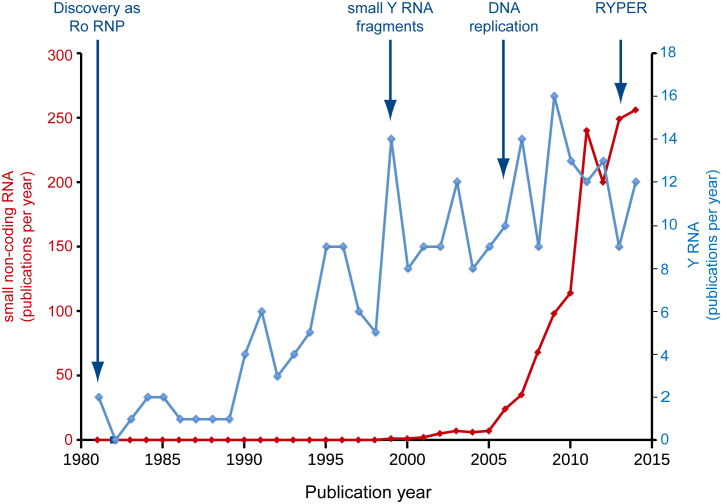
Approximate number of publications per year relating to small non-coding RNA (left *y*-axis) and Y RNA (right *y*-axis), respectively. Data are the number of hits on a Thomson Reuters Web of Science topic search for the phrases ‘small non-coding RNA’ and ‘Y RNA’ (including ‘YRNA’, ‘RoRNP’, ‘Ro RNP’ and ‘Y5 RNA’ with white space characters).

**Fig. 3:**
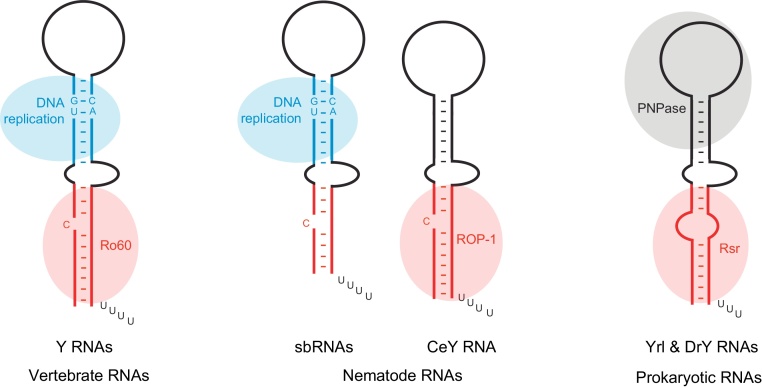
Modular structure of prokaryotic and eukaryotic Y RNAs. The overall secondary structures of the indicated RNAs are shown schematically and are based on consensus structures. Secondary structures in the divergent loop domains are omitted for clarity. RNA motifs acting as binding sites for Ro60 protein orthologues are shown in red. RNA motifs required for DNA replication are highlighted in blue, and interaction sites for the prokaryotic exonuclease PNPase are highlighted by a grey circle. See main text for references. (For interpretation of the references to color in this legend, the reader is referred to the web version of the article.)

Since their discovery in 1981, the Y RNA field has grown considerably, concomitant with the rise in number of independent cellular roles associated with Y RNAs (Fig. 2). Biochemical functions of Y RNAs have been mapped to distinct structural domains of the Y and sbRNAs. Therefore, to reconcile this diverse range of functions, a concept of modular structure and evolution of these RNAs is now becoming apparent (Fig. 3).

Initially, investigations into Y RNA function focussed on the conserved binding sites for Ro60 and La proteins, which are required for Y RNAs to associate with these proteins to form RoRNPs (Chen and Wolin, 2004). RoRNPs are currently implicated in RNA processing and quality control (Hall et al., 2013, Wolin et al., 2012). The precise cellular function of RoRNPs is not yet clear and furthermore they are not essential as deletion mutants of Ro60 are viable. The highly conserved binding sites for Ro60 and La proteins are present in the lower stem and polyuridine tail domains of Y RNAs, respectively (Fig. 1).

More recently Y RNAs were shown to be essential factors for the initiation step of chromosomal DNA replication in human cell nuclei (Christov et al., 2006, Krude et al., 2009). Strikingly, the essential cellular function of Y RNAs in DNA replication is conserved in vertebrates and nematodes, since functional inactivation of Y RNAs in *Xenopus laevis* and *Danio rerio* embryos, or of sbRNAs in *Caenorhabditis elegans* leads to abrogation of DNA replication, cell cycle arrest and embryonic lethality (Collart et al., 2011, Kowalski et al., 2015). In contrast to Ro60 and La binding, this essential function resides in the upper stem domain of these RNAs (Gardiner et al., 2009, Kowalski et al., 2015, Wang et al., 2014).

The loop domain of Y RNAs is diverse in sequence and has been reported to bind several different proteins, including nucleolin, polypyrimidine tract-binding protein (PTB) and zipcode binding protein 1 (ZBP1) (Köhn et al., 2013) (Fig. 1). The roles of the interactions with these proteins are unclear, but it has been suggested that they could modulate the subcellular localisation of Ro60 (Sim and Wolin, 2011), and also confer specialised cellular functions by binding preferentially to specific Y RNAs (Hogg and Collins, 2007, Langley et al., 2010). Consistent with its wide array of binding proteins, the loop domain has been implicated in modulating the association of Y RNAs with subnuclear chromatin domains (Zhang et al., 2011).

Finally, small RNA fragments derived from Y RNAs become enriched in apoptotic cells, possibly as result of apoptotic degradation processes (Rutjes et al., 1999). However, recent high-throughput RNA sequencing approaches have now established that small Y RNA fragments are also highly abundant in cells, tissues and body fluids of humans and mammals, as well as in a range of tumours (Dhahbi et al., 2014, Meiri et al., 2010, Nicolas et al., 2012, Vojtech et al., 2014). Therefore, these Y RNA-derived fragments are now of clinical interest and have attracted much recent attention as potential biomarkers for disease.

In this review, we discuss the biochemical and cellular functions of Y RNAs, as well as their evolutionary conservation. The reader is referred to earlier reviews that have focused on individual aspects of Y RNA and RoRNP biology (Chen and Wolin, 2004, Hall et al., 2013, Köhn et al., 2013, Krude, 2010, Pruijn et al., 1997, Verhagen and Pruijn, 2011, Wolin et al., 2012, Wolin et al., 2013). Here, we present a current integrated view of Y RNA function, focussing on the modular domain structure of Y RNAs, which can mediate the assembly of distinct Y RNPs under different cellular conditions and contexts.

## 2 Y RNAs form RoRNPs that are involved in the regulation of RNA stability and cellular stress responses

Y RNAs were first identified as non-coding RNAs bound by the Ro60 protein, a common 60 kDa antigen detected by antibodies from patients with the autoimmune diseases Systemic Lupus Erythematosus and Sjögren's syndrome (Lerner et al., 1981). Ro60 is conserved in vertebrates and homologues have been identified in most metazoa, and also in ∼5% of sequenced bacterial genomes, including *D. radiodurans* and *Salmonella* (Sim and Wolin, 2011, Wolin et al., 2013).

In all organisms studied, orthologues of Ro60 protein bind to Y RNAs, or Y RNA-like (Yrl) non-coding RNAs to form RoRNPs (Chen et al., 2013, Chen et al., 2014) (Fig. 3). In vertebrates, the Ro60 binding site on the lower stem domain of Y RNAs is well characterised and comprises a seven-base-pair helix, a single bulged cytidine and a three-nucleotide bulge on the opposite strand (Pruijn et al., 1991, Wolin and Steitz, 1984) (Fig. 1). In vertebrates, Ro60-binding to the Y RNA lower stem is dependent on both RNA sequence-specific interactions and shape complementarity (Stein et al., 2005). The two bulges in the lower stem of Y RNAs distort its helical structure, making the major groove of the RNA accessible to the amino side chains of Ro60 (Green et al., 1998). Mutations in the Y RNA lower stem that remove either bulge, or change the conserved nucleotide sequence, abolish Ro60 binding (Green et al., 1998, Pruijn et al., 1991). Finally, X-ray crystallography studies show that Ro60 is toroidal in shape, binds Y RNAs on its outer surface and contains a positively charged central channel that can accommodate single-stranded, but not double-stranded RNA (Stein et al., 2005). Immunoprecipitation experiments have shown that in the nematode *C. elegans* the Ro60 orthologue protein, ROP-1, binds only a single major RNA, termed CeY RNA (Van Horn et al., 1995). Although a recent study suggested that the sbRNA CeN72 interacts with ROP-1 in a gel shift assay *in vitro* (Xiao et al., 2012), neither CeN72 nor any of the other 17 *C. elegans* sbRNAs were identified in ROP-1 immunoprecipitates from worm extracts (Van Horn et al., 1995). Consistent with these observations, it was suggested that the CeY RNA is an outlier of the sbRNA family and that it may have undergone a functional specialisation towards RoRNP-related functions in nematodes (Boria et al., 2010). In prokaryotes, the Ro60 orthologue protein Rsr binds to the lower stem of DrY RNA in *D. radiodurans* and Yrl RNAs in *Salmonella* and *M. smegmatis* (Chen et al., 2013, Chen et al., 2014).

La protein is a 50 kDa auto-antigen found complexed with a subset of RoRNPs (Hendrick et al., 1981). La is required for accurate and efficient termination of RNA polymerase III transcription, and binds to the 3′ polyuridine tail of newly synthesised RNAs in the nucleus (Stefano, 1984). While most mature RNA transcripts lose their polyuridine tail, Y RNAs retain theirs (Fig. 1), and so can maintain association with La. La is implicated in the nuclear retention of Y RNAs and protecting RNAs from exonucleolytic cleavage (Wolin and Cedervall, 2002).

RoRNPs are currently implicated in non-coding RNA quality control, RNA stability and in cellular responses to stress in several organisms (Sim and Wolin, 2011). Ro60 binds aberrant non-coding RNAs such as misfolded 5S rRNA or U2 snRNA in a range of species, including *X. laevis*, *C. elegans* and *Mus musculus* (Chen et al., 2003, Labbe et al., 1999a, Labbe et al., 1999b, O’Brien and Wolin, 1994). *C. elegans* lacking ROP-1 are viable, but these deletion strains show defects in dauer larvae formation, an alternative developmental stage induced by starvation or stress that allows them to survive unfavourable environmental conditions (Labbe et al., 1999b, Labbe et al., 2000). Furthermore, mouse cells and *D. radiodurans* upregulate and accumulate RoRNPs in response to ultraviolet (UV) irradiation and both cell types lacking Ro60 have decreased survival following UV irradiation (Chen et al., 2000, Chen et al., 2003, Xue et al., 2003). In *D. radiodurans*, the Ro60 orthologue protein Rsr also has a role in heat-stress-induced rRNA maturation and starvation-induced rRNA decay (Chen et al., 2007, Wurtmann and Wolin, 2010). Ro60 binding to misfolded non-coding RNAs is not largely sequence specific, indicating that Ro60 could potentially bind a wide range of RNAs (Fuchs et al., 2006). It has therefore been proposed that RoRNPs function as cellular stress sensors, which scavenge and process aberrant non-coding RNAs that fail to associate with their cognate RNA-binding proteins (Fuchs et al., 2006, Hogg and Collins, 2007).

Genetic deletion studies have established that Ro60 protein and its orthologues ROP-1 and Rsr in nematodes and prokaryotes, respectively, are not essential for cell proliferation, or the viability and development of the unperturbed organism (Chen et al., 2000, Chen et al., 2003, Labbe et al., 1999b, Xue et al., 2003). However, these deletions of Ro60 and its orthologues resulted in significant reductions in the levels of soluble eukaryotic or prokaryotic Y RNAs. It has therefore been concluded from these studies that Ro60 proteins play a functional role in the stability of their associated Y RNAs.

The precise role of Y RNAs in vertebrate RoRNPs has proved controversial. Structural and biochemical studies have shown that misfolded RNAs insert through the Ro60 cavity and also bind to the Ro60 outer surface at a region that partially overlaps with the Y RNA-binding domain (Fuchs et al., 2006, Stein et al., 2005). Since Y RNAs bind Ro60 in a sequence-specific manner and with higher affinity than misfolded RNAs, it has been suggested that a bound Y RNA could sterically bock misfolded RNA binding to Ro60 (Fuchs et al., 2006, Stein et al., 2005). However, all four human Y RNAs bind to Ro60 and La, but only hY5 RNA co-purified with a common target of RNA quality control, 5S rRNA, *via* ribosomal protein L5 (Hogg and Collins, 2007). This work suggested that hY5-RoRNPs interact with target 5S-L5 RNPs, so that specific Y RNAs might themselves modulate the recruitment of misfolded or variant non-coding RNAs to RoRNPs. A unified model has been proposed in which Y RNAs can both positively and negatively regulate the target specificity of non-coding RNA quality control mediated by Ro60 (Hogg and Collins, 2007).

Interestingly, recent studies in *D. radiodurans* have demonstrated that prokaryotic Y RNAs regulate both access of the Ro60 orthologue protein Rsr to RNA substrates and also recruit exonucleases involved in their maturation or degradation (Chen et al., 2007, Chen et al., 2013). In *D. radiodurans*, the prokaryotic DrY RNA tethers Rsr to the exoribonuclease polynucleotide phosphorylase (PNPase), forming RYPER (Ro60/Y RNA/PNPase exoribonuclease RNP), an RNA degradation complex that cleaves structured RNAs (Chen et al., 2013, Wolin et al., 2013). In this specialised RoRNP, the Y RNA acts as a scaffold linking Rsr with PNPase. It also serves as a gate mediating the entry of single-stranded RNA substrates into the PNPase cavity, thereby modulating the substrate specificity of the enzyme and increasing the effectiveness or RYPER (Chen et al., 2013). It remains to be seen whether metazoan RoRNPs with their Y RNAs are also involved in nucleolytic degradation of target RNAs.

Taken together, in these past three-and-a-half decades of research on the RoRNP, a substantial body of structural and functional data has accumulated that supports a functional role for Y RNAs in RNA stability and quality control. This allocation of Y RNA function comes from a sequence- and structure-specific association of pro- and eukaryotic Y RNAs with members of the Ro protein family. In all cases, this interaction, and thus Y RNA involvement in a functional role of the resulting RNP, occurs *via* the evolutionarily conserved lower stem of the Y RNAs (Fig. 3).

## 3 Y RNAs are essential factors for the initiation of chromosomal DNA replication

The first direct and essential cellular function that has been experimentally demonstrated for Y RNAs is their involvement in the initiation of chromosomal DNA replication (Christov et al., 2006, Christov et al., 2008, Collart et al., 2011, Gardiner et al., 2009, Krude et al., 2009). In an unbiased approach, Y RNAs were purified by biochemical fractionation of a human cell extract as an activity that is essential for the reconstitution of chromosomal DNA replication in a cell-free system (Christov et al., 2006). This *in vitro* system uses nuclei that are prepared from late G1 phase human cells. Semi-conservative DNA replication initiates and subsequently elongates in these nuclei upon the addition of cytosolic extract from proliferating human cells (Krude, 2000, Krude et al., 1997). During its step-wise purification, one particular cytosolic replication factor maintained DNA replication activity over several biochemical steps that enriched for poly-anionic factors. Surprisingly, this approach led to the purification of non-coding Y RNAs as the relevant factor and not of a protein, which was expected at the time (Christov et al., 2006). In support of these gain-of-function experiments, specific degradation of Y RNAs from unfractionated cytosolic cell extract abrogates the initiation step of DNA replication (Christov et al., 2006, Gardiner et al., 2009, Krude et al., 2009). DNA replication can then be restored by addition of non-targeted individual human or vertebrate Y RNAs, but not of other small non-coding RNAs such as 5S ribosomal RNA or U2 RNA. Therefore, vertebrate Y RNAs are required specifically for DNA replication, and they function redundantly with each other in this system. Single molecule analysis of Y RNA depletion and reconstitution experiments provided detailed and direct evidence that Y RNAs are required for the initiation step of DNA replication, leading to the establishment of new DNA replication forks on human chromosomal DNA (Krude et al., 2009). In contrast, Y RNAs are not required for the elongation of existing DNA replication forks, and are thus not involved in the DNA copying mechanism as such (Krude et al., 2009). From these experiments, it has also become clear that Y RNAs do not fulfil this important function in isolation, but require interaction with other DNA replication proteins that are also present in the extract (Christov et al., 2006).

Systematic mutagenesis of vertebrate Y RNAs identified that the upper stem domain is necessary and also sufficient for Y RNA function in the initiation of DNA replication (Gardiner et al., 2009). The upper stem domain is present in all vertebrate Y RNAs and can thus explain the functional redundancy of vertebrate Y RNAs. This domain contains a highly conserved central GUG–CAC nucleotide sequence motif (Gardiner et al., 2009) (Fig. 1). A recent structure analysis of the upper stem by nuclear magnetic resonance (NMR) and far-UV circular dichroism (CD) spectroscopy provided evidence that this domain adopts a locally destabilised A-form helix under physiological conditions in solution (Wang et al., 2014). The helix is stabilised by two flanking G–C base pairs, but the central section around the highly conserved G–C base pair (*i.e.* the upper one of the GUG–CAC motif) is unstable and the accessible bases may thus be involved in specific interactions of this domain with as yet unknown proteins (Wang et al., 2014). Mutations in this sequence motif abrogate the initiation activity of the Y RNA, concomitant with structural perturbation of the upper stem domain (Gardiner et al., 2009, Wang et al., 2014). Conversely, an insertion of the upper stem domain into a similarly folded, but inactive, backbone of a synthetic RNA results in the full activation of this previously inert RNA as a DNA replication initiation factor (Gardiner et al., 2009). Furthermore, the lower stem and loop domains of Y RNAs are dispensable as they can be entirely removed from the RNA without loss of DNA replication initiation function (Gardiner et al., 2009).

Importantly, neither Ro60 or La proteins, nor their binding sites in vertebrate Y RNAs, are required for the initiation of DNA replication. Immunoprecipitation experiments indicate that ∼50% of Y RNAs in human cell extracts are present outside Ro60 and La RNPs (Langley et al., 2010). Immunodepletion of Ro60 and La RNPs from human cytosolic extracts does not inhibit DNA replication in human cell nuclei (Langley et al., 2010). Furthermore, addition of recombinant purified Ro60 or La proteins has no effect on DNA replication *in vitro* (Langley et al., 2010). Deletion of Ro60 and La binding sites on the lower stem domain of vertebrate Y RNAs does not inhibit the DNA replication initiation activity of the mutant Y RNAs (Christov et al., 2006, Gardiner et al., 2009). These findings indicate that Y RNAs mediate the initiation of DNA replication independently of RoRNPs. Consistent with these *in vitro* findings, genetic knockout of Ro60 in various organisms has no effect on DNA replication or viability (Chen et al., 2000, Labbe et al., 1999b, Xue et al., 2003).

Vertebrate Y RNAs are also essential for initiation of DNA replication *in vivo*. Disruption of Y RNAs by RNAi in proliferating vertebrate cells in culture blocks DNA replication and cell proliferation (Christov et al., 2006, Christov et al., 2008, Collart et al., 2011). Interestingly, transfection of a synthetic small double-stranded RNA derived from the upper stem of hY1 RNA overcomes this inhibition (Gardiner et al., 2009), demonstrating that its replication function is indeed responsible for the *in vivo* phenotype. Furthermore, functional inactivation of Y RNAs by microinjection of antisense morpholino oligonucleotides (MOs) into *D. rerio* or *X. laevis* embryos leads to DNA replication inhibition, arrested development and early embryonic death (Collart et al., 2011), which occurs right after the mid-blastula transition (MBT) (Langley et al., 2014). Consistent with a functional role in DNA replication and cell proliferation, Y RNAs are over-expressed in human solid tumours, when compared with the corresponding healthy tissues (Christov et al., 2008). Taken together, these observations establish that Y RNA function is built on a modular structure of the overall full-length RNA. The essential function for chromosomal DNA replication in vertebrates can be ascribed to the short upper stem domain of the vertebrate Y RNAs.

Recently, a family of related small non-coding RNAs, termed stem-bulge RNAs (sbRNAs), was identified in nematode worms (Aftab et al., 2008, Boria et al., 2010, Deng et al., 2006), and an sbRNA was also described for the silkworm, *B. mori* (Duarte et al., 2015). The genome of *C. elegans* contains at least 18 sbRNA genes, in addition to the related aforementioned CeY RNA gene, each with a putative RNA polymerase III promoter, with many sbRNA genes also present in other nematode species (Boria et al., 2010). A computational analysis based on nucleotide sequence and structural motifs suggested that sbRNAs might be homologues of vertebrate Y RNAs, the previously described CeY RNA being an outlier of this group because of a lower sequence conservation compared to the other sbRNAs (Boria et al., 2010). Y RNAs and sbRNAs share an overall stem-loop structure containing double-stranded upper and lower stem domains, as well as a single-stranded internal loop (Boria et al., 2010). The upper stem domain of sbRNAs, like vertebrate Y RNAs, contains a highly conserved A/GUG–CAC/U motif (Boria et al., 2010, Kowalski et al., 2015) (Fig. 3). We have recently shown that sbRNAs from several nematode species can functionally substitute for vertebrate Y RNAs and support the initiation of chromosomal DNA replication *in vitro*, whereas CeY RNA does not (Kowalski et al., 2015). Importantly, the initiation activity of full-length sbRNAs was dependent on the upper stem domain and intriguingly, also on a conserved UUAUC motif in the loop domain, which is also present in vertebrate Y RNAs (Kowalski et al., 2015). Furthermore, functional inhibition of sbRNAs in *C. elegans* resulted in DNA replication defects and lethality during early embryogenesis (Kowalski et al., 2015). Collectively, these findings indicate that sbRNAs are functional homologues of vertebrate Y RNAs. To date, candidate Y RNAs or sbRNAs have not been identified in plants or fungi and it therefore remains to be seen to what extent the regulation of DNA replication by small stem-loop RNAs has been conserved during eukaryotic evolution.

Y RNAs have also been reported in prokaryotes and some other isolated eukaryotic species (Chen et al., 2000, Chen et al., 2013, Chen et al., 2014, Mosig et al., 2007, Perreault et al., 2007, Van Horn et al., 1995). The Y RNAs from *C. elegans* (CeY RNA), *B. floridae* (BfY RNA) and *D. radiodurans* (DrY RNA) do not have sequence similarity to vertebrate Y RNAs in the upper stem domain and they are unable to substitute for vertebrate Y RNAs in DNA replication assays *in vitro* (Gardiner et al., 2009). These results indicate that these non-vertebrate Y RNAs do not fulfil the role of vertebrate Y RNAs in DNA replication. Furthermore, *D. radiodurans* or *C. elegans* with a deletion in their respective genes coding for DrY and CeY RNA are viable, so these RNAs are not essential for DNA replication and viability of the organism (Boria et al., 2010, Chen et al., 2007). Thus, in *C. elegans* a large family of sbRNAs is found, at least some of which function in DNA replication and do not appear to bind Ro60, whilst the divergent CeY RNA binds to Ro60 and does not function in DNA replication (Fig. 3).

The mechanism of Y RNA function in the initiation step of DNA replication in vertebrates is not yet clear, although several key features are emerging. Y RNAs interact biochemically with several DNA replication initiation proteins, including the origin recognition complex ORC, and initiation proteins Cdc6, Cdt1 and DUE-B (Collart et al., 2011, Zhang et al., 2011). In contrast, hY RNAs do not interact biochemically with DNA replication fork proteins including the DNA helicase subunits MCM2-7, GINS complex, primase, or DNA polymerases (Zhang et al., 2011). These biochemical interactions would therefore suggest a functional interaction between Y RNAs and the protein machinery of the DNA replication initiation complex. Using fluorescently-labelled hY RNAs, it was shown that hY RNAs also associate dynamically with unreplicated chromatin in G1 phase nuclei *in vitro*, where they co-localise with several DNA replication proteins on chromatin before the initiation of DNA replication, including ORC, Cdt1, MCM2 and Cdc45 (Zhang et al., 2011). In *X. laevis*, Y RNA binding to chromatin occurs only after the MBT and is ORC-dependent (Collart et al., 2011). Once DNA replication initiates in a Y RNA-dependent manner, Y RNAs are locally displaced from these initiation sites, and they are consequently absent from the sites of ongoing DNA synthesis in these nuclei (Zhang et al., 2011). It has therefore been suggested that Y RNAs could function in a ‘catch-and-release’ mechanism on chromatin in human cells (Zhang et al., 2011), which is consistent with the original ‘licensing factor’ model of Blow and Laskey (Blow et al., 1987, Laskey et al., 1981). Future experiments are needed to test this hypothesis and resolve the underlying molecular mechanism of Y RNA function during the initiation of chromosomal DNA replication. Questions to address will include whether or not the upper stem domain of Y RNAs hybridises with other nucleic acids in order to execute its essential function; which are the functionally essential interacting DNA replication proteins and whether Y RNAs activate these proteins or inactivate any potential repressors.

## 4 Y RNA localisation

In eukaryotes the biogenesis of Y RNAs begins in the nucleus, as RNA polymerase III transcription is terminated. As with other RNA polymerase III transcripts such as tRNAs or pre-miRNAs, Y RNAs can be exported to the cytoplasm, or like U snRNAs, they can remain in the nucleus after transcription.

There are conflicting reports on the relative distribution of Y RNAs in the nucleus and cytoplasm of vertebrate cells, likely in part due to different methodologies used (Hall et al., 2013, Pruijn et al., 1997, Zhang et al., 2011). Early enucleation and cell fractionation experiments revealed that Y RNAs were predominantly, or even exclusively, cytoplasmic in cultured mammalian cells and *X. laevis* oocytes (O’Brien et al., 1993, Peek et al., 1993, Simons et al., 1994). One study reported more recently that in human and mouse cells, h/mY1, h/mY3 and hY4 RNAs are found in the cytoplasm, whereas hY5 RNA localises to the nucleus (Gendron et al., 2001). *In situ* hybridisation and ultrastructural analysis by electron microscopy, however, showed that Y RNAs are present at discrete sites in both the nucleus and cytoplasm of cultured human cells (Farris et al., 1997, Matera et al., 1995). Furthermore, in proliferating human cells hY1, hY3 and hY5 RNAs also localise to the edge of nucleoli (the perinucleolar compartment) and co-localise with PTB at these sites in the cell nucleus (Matera et al., 1995). Using fluorescently-labelled hY RNAs it was shown that all four hY RNAs bind chromatin in G1 phase nuclei dynamically from a soluble pool as the nuclei enter S phase *in vitro* (Zhang et al., 2011). While hY1, hY3 and hY4 co-localise with each other and associate mostly with early-replicating euchromatin, hY5 is enriched in nucleoli (Zhang et al., 2011). The loop domain of hY RNAs modulates this differential association with chromatin as mutant hY RNAs lacking this domain bind to chromatin indiscriminately (Zhang et al., 2011). It remains to be seen if this dynamic chromatin association of Y RNAs is important for their function in the initiation of DNA replication, and in how far it is regulated during the cell cycle. In any case, there is now accumulating evidence that Y RNAs are present in both the nuclear and cytoplasmic fractions of eukaryotic cells, and that their relative abundance in these cell compartments most likely reflects the methodologies used for study and/or the physiological state of the cells.

The export pathways used by Y RNAs are also becoming increasingly well understood. Y RNA export is dependent on the small GTPase Ran, indicating that exportins likely serve as transport receptor proteins for Y RNAs (Rutjes et al., 2001). The lower stem of Y RNAs resembles a dsRNA mini-helix present in other exportin-5 substrates and exportin-5 was shown to associate in a complex with hY1 RNA and RanGTP (Gwizdek et al., 2001, Gwizdek et al., 2003). Furthermore, deletion of the lower stem of hY1 RNA results in defective nuclear export of the mutant RNA in *Xenopus* oocytes (Rutjes et al., 2001). Therefore, it seems likely that Y RNAs are exported from the nucleus in an exportin-5-dependent manner. Direct mechanistic evidence of a re-import pathway of Y RNAs back into the nucleus has not been identified to date.

Ro60 binding protects Y RNAs from exonucleolytic degradation and is required for stable accumulation of Y RNAs in a range of species (Chen and Wolin, 2004). It has been proposed that one function of mammalian Y RNAs is to regulate the subcellular localisation of Ro60 (Sim and Wolin, 2011). Ro60 is present in both the nucleus and cytoplasm of cells and it has been shown that Y RNAs can influence this distribution (Sim and Wolin, 2011). In mouse cells, Y RNA binding to Ro60 occludes a nuclear localisation signal on the Ro60 surface, thereby retaining RoRNPs in the cytoplasm (Sim et al., 2009). Another study has demonstrated that Ro60 binding is a prerequisite for efficient nuclear export of Y RNAs in *X. laevis* oocytes (Simons et al., 1996).

The intracellular localisation of Y RNAs changes under conditions of cellular stress (Chen and Wolin, 2004). Both Ro60 and Y RNAs accumulate in the nucleus after UV irradiation or oxidative stress in several species (Chen et al., 2000, Chen et al., 2003, Sim et al., 2009, Sim et al., 2012). This is consistent with a role for nuclear Ro60-Y RNA complexes in cellular stress responses. However, it could also arise from stress-induced inhibition of the RanGTP gradient, resulting in defective nuclear export and thus nuclear accumulation (Köhn et al., 2013).

Mammalian Y RNAs can also be selectively packaged into viruses. This has been demonstrated for the human immunodeficiency virus type 1 (HIV-1) and Moloney murine leukaemia virus (Garcia et al., 2009, Wang et al., 2007). This process does not require Ro60 binding and likely occurs during early stages of Y RNA biogenesis when nascent Y RNAs are present in the nucleus (Wang et al., 2007). It is currently unknown whether Y RNAs are involved in retroviral function.

Unlike vertebrate Y RNAs, the intracellular localisations of nematode sbRNAs and CeY RNA have not yet been investigated. It therefore remains to be seen whether the functional homology between vertebrate Y RNAs and nematode sbRNAs is also reflected in a similar distribution of these nematode sbRNAs in the nuclear and cytoplasmic fractions of the cell.

## 5 Y RNA loop domain-binding proteins

Recently, several novel Y RNA-binding proteins have been identified in eukaryotic cells. It was shown using gel filtration that Y RNPs present in human cell extracts range in size from 150 to 550 kDa, indicating that Y RNAs can associate with multiple proteins simultaneously (Fabini et al., 2000). However, apart from the canonical Y RNA-binding proteins Ro60 and La, the interactions between other cellular proteins and Y RNAs are currently poorly characterised.

Several proteins interact with Y RNAs through the loop domain, which is the least conserved domain in Y RNAs; it is heterogeneous in structure and varies in nucleotide sequence between individual Y RNAs (Fig. 1) (Farris et al., 1999, Teunissen et al., 2000). The loop domains of hY1, hY3 and hY5 RNAs are pyrimidine-rich, and in hY1 and hY3 RNAs, contain stretches of poly-pyrimidine sequences. The loop domains of Y RNAs can interact with a different set of proteins to form distinct Y RNPs (Bouffard et al., 2000, Fabini et al., 2001, Hogg and Collins, 2007). The loop domain may therefore specialise individual Y RNAs for specific cellular functions (Hogg and Collins, 2007).

In human cells, several Y RNA-loop-binding proteins have been identified, including nucleolin, PTB/hnRNP I, hnRNP K and ZBP1 (Köhn et al., 2013). Nucleolin binds pyrimidine-rich stretches in the loop domain of Y RNAs in human cells, and while it preferentially associates with hY1 and hY3, it is present in stable cytosolic RNPs with all four hY RNAs (Fabini et al., 2001, Langley et al., 2010). Nucleolin is involved in many metabolic processes, including rRNA processing, ribosome biogenesis and nucleo-cytoplasmic transport (Ginisty et al., 1999). Cytosolic nucleolin RNPs are distinct from Ro60 and La RNPs and immunodepletion of nucleolin RNPs from cytosolic extracts does not inhibit DNA replication initiation in human cell nuclei (Langley et al., 2010). Therefore the interaction between Y RNAs and soluble nucleolin is not required for Y RNA function in this process (Langley et al., 2010).

PTB and hnRNP K bind preferentially to poly-pyrimidine tracts in the loop domains of hY1 and hY3 RNAs, like nucleolin, but their association with hY4 and hY5 RNAs has not been detected (Fabini et al., 2001, Fouraux et al., 2002). Efficient binding of PTB and hnRNP K to Y RNAs also likely requires La, because deletion of the La binding site significantly decreases binding of both proteins to the RNA (Fabini et al., 2001). PTB and hnRNP K are both involved in several aspects of RNA processing and are thought to shuttle between the nucleus and the cytoplasm (Krecic and Swanson, 1999). PTB, hnRNP K and La function as RNA chaperones *in vitro* and mediate RNA folding without a requirement for ATP consumption (Belisova et al., 2005). Since Y RNA binding to these proteins inhibits their RNA chaperone activity, it has been proposed that Y RNAs mediate the transport of hnRNP K, PTB and La to specific targets, before releasing the proteins to execute their function (Belisova et al., 2005). ZBP1 interacts with mouse Y1 and Y3 RNAs *via* their loop domains (Köhn et al., 2013, Sim et al., 2012). Depletion of ZBP1 results in accumulation of Y3 RNA in mouse cell nuclei, indicating that ZBP1 is involved in nuclear export of Y RNPs (Sim et al., 2012).

All four human Y RNAs associate with the antiviral cytidine deaminase APOBEC3G, which is also a component of Ro60 and La RNPs (Chiu et al., 2006, Gallois-Montbrun et al., 2008). Although the function of these Y RNPs is unknown, it has been speculated that APOBEC3G could be involved in RNA editing of Y RNAs to modulate their function (Chiu et al., 2006, Köhn et al., 2013).

There are also several proteins that bind preferentially or exclusively to Y5 RNAs, indicating a specialised role for this Y RNA. RoRNP binding protein I (RoBPI) mainly associates with hY5 RNA in human cells (Bouffard et al., 2000) but also binds hY1 and hY3 RNAs (Hogg and Collins, 2007). RoBPI is a DNA- and RNA-binding protein involved in several nuclear processes, such as transcription and RNA splicing (Page-McCaw et al., 1999). In addition, Interferon-induced protein with tetratricopeptide repeats 5 (IFIT5) only interacts with hY5 RNA (Hogg and Collins, 2007). Ribosomal protein L5 also interacts specifically with hY5 RNA (Hogg and Collins, 2007). The L5 protein forms complexes with 5S rRNA (Steitz et al., 1988) and Y5 RNA also associates with 5S rRNA, with a strong preference for a misfolded variant (Hogg and Collins, 2007). Together with the observed enrichment of hY5 in nucleoli (Zhang et al., 2011), these results indicate that hY5 RNA could be involved in rRNAs biogenesis (Hogg and Collins, 2007).

The identification of these non-canonical Y RNA-binding proteins, many of which show preferential binding to specific Y RNAs, indicates that Y RNAs are likely incorporated into multiple, distinct RNPs to carry out specialised functions (Bouffard et al., 2000, Fabini et al., 2001, Hogg and Collins, 2007, Langley et al., 2010). It is also possible that Y RNAs sequester multiple cellular proteins until they are needed, for example in response to stress (Köhn et al., 2013). In addition, the localisation of Y RNAs is also influenced by these interacting proteins (Köhn et al., 2013, Zhang et al., 2011). In conclusion, the loop domains of eukaryotic Y RNAs attract many divergent binding proteins and further work is required to understand mechanistically any potential functional roles for the resulting different Y RNP complexes.

## 6 Y RNA-derived small RNAs

Recently, a plethora of deep sequencing studies in eukaryotes have identified small RNA fragments derived from longer RNAs (Rother and Meister, 2011, Tuck and Tollervey, 2011). Accumulating evidence suggests that these RNA fragments, while derived from pre-existing small non-coding RNAs, are themselves physiologically relevant in both healthy and diseased cells (Dhahbi, 2014, Hall and Dalmay, 2013).

High levels of Y RNA-derived small RNAs (YsRNAs) of 22–36 nucleotides are produced in apoptotic cells (Rutjes et al., 1999). Immunoprecipitation experiments have revealed that these YsRNAs are bound to Ro60 and La proteins, suggesting that the binding sites of these proteins in the lower stem domain of Y RNAs are protected from the nucleolytic degradation process (Rutjes et al., 1999). It remains to be seen whether the upper stem domain of Y RNAs, which is required for Y RNA functionality in DNA replication and cell proliferation, is actively targeted during apoptosis.

YsRNAs are also detected in proliferating cells, both cancerous and non-cancerous, at levels similar to that of known miRNAs (Nicolas et al., 2012). YsRNAs are also found in the brain, retina and other healthy mammalian tissues, as well as in a range of tumours (Chen and Heard, 2013, Meiri et al., 2010, Verhagen and Pruijn, 2011, Yamazaki et al., 2014). Some of these YsRNAs were initially mis-annotated as a novel type of miRNA generated by the processing of full-length Y RNAs (Meiri et al., 2010, Verhagen and Pruijn, 2011). However, it has subsequently been shown that YsRNA biogenesis is independent of the canonical miRNA biogenesis pathway. YsRNAs do not associate with Argonaute proteins (Chen and Heard, 2013, Nicolas et al., 2012), and the generation of YsRNAs appears to be independent of Dicer (Langenberger et al., 2013). Furthermore, in contrast to miRNAs, YsRNAs do not have gene silencing activity in the luciferase reporter assay (Meiri et al., 2010). The role of these intracellular YsRNAs is currently unknown.

YsRNAs, along with tRNA fragments, have been recently identified as highly abundant small RNAs circulating in the blood of humans and other mammals (Dhahbi, 2014). Circulating YsRNAs of 25–33 nucleotides are present in human blood in multiple forms, including within vesicles and as cell-free RNP complexes of 100–300 kDa (Dhahbi, 2014, Dhahbi et al., 2013, Dhahbi et al., 2014). These YsRNAs are derived from the 5′ and 3′ termini of full-length Y RNAs by cleavage within the internal loop domain (Dhahbi et al., 2013, Dhahbi et al., 2014). The levels of these 5′ and 3′-Y RNA and 5′-tRNA-derived fragments were found to be significantly different in a group of breast cancer patients compared to healthy individuals, suggesting that these fragments may have some diagnostic value as cancer biomarkers (Dhahbi et al., 2014). Interestingly, many of the Y RNAs from which the fragments are derived have previously been annotated as Y RNA pseudogenes, and therefore not thought to be expressed or processed (Dhahbi et al., 2013, Dhahbi et al., 2014). Since YsRNAs have no known functions, the significance of the pseudogene expression is unclear.

YsRNAs and full-length Y RNAs have also been detected in vesicles released by mouse immune cells (Nolte-’t Hoen et al., 2012) and YsRNAs comprise a substantial fraction of the RNA component of exosome vesicles present in human semen (Vojtech et al., 2014). The function of these extracellular YsRNAs is currently unknown and whilst it has been speculated that YsRNAs are specifically processed and secreted as part of an as yet undefined signalling process (Dhahbi, 2014), these RNA fragments could alternatively just be passive, stable degradation products of highly abundant cellular Y RNAs. However, it is an intriguing possibility that small RNAs could mediate intercellular physiological signals (Chen et al., 2012, Hoy and Buck, 2012, Sarkies and Miska, 2014, Valadi et al., 2007) and therefore the clinical potential of YsRNAs as diagnostic biomarkers or blood-delivered therapy targets remains an open and exciting possibility.

## 7 Conclusions

Y RNAs are small non-coding RNAs involved in a range of cellular processes, including DNA replication, RNA stability and cellular stress responses. The modular domains of Y RNAs mediate their distinct cellular roles (Fig. 1, Fig. 2, Fig. 3). The upper stem domain of vertebrate Y RNAs and homologous nematode sbRNAs is essential for the initiation of chromosomal DNA replication. The lower stem is required for Ro60 binding and hence is involved in stress responses, RNA surveillance, stability control and RoRNP-mediated RNA degradation across several eukaryotic and prokaryotic species. Furthermore, the evolutionary conservation of the structure and nucleotide sequence of the upper and lower stem domains is tightly correlated to the conservation of Y RNA functions across species, indicative of high selective pressure on the Y RNA domains. The loop domain, although part of the conserved overall secondary structure of Y RNAs, is highly varied in its nucleotide sequence. This might facilitate functional sub-specialisation of different Y RNA molecules within the same species. This RNA module-based strategy might provide a way of regulating and separating important cellular functions of the small non-coding Y RNAs. With the recent emergence of an expansive landscape of pervasive transcription and non-coding RNAs in mammalian cells (Clark et al., 2011, Iyer et al., 2015), this principle also has direct implications for non-coding RNA evolution. As seen with Y and sbRNAs across eukaryotes and prokaryotes, each modular RNA domain can evolve separately and thus pave the way for functional divergence and specialisation of non-coding RNAs (Fig. 3). Since the discovery of Y RNAs in 1981, much progress has been made in elucidating their binding proteins, evolutionarily conservation and important cellular roles. However, many key questions remain, which provide opportunity for exciting further growth in the field of non-coding Y RNAs (Box 1).

## Box 1

**Outstanding questions**

• How do Y RNAs regulate the initiation of DNA replication in vertebrates?
• Is the requirement for small stem-loop RNAs in the initiation of chromosomal DNA replication conserved beyond vertebrates and nematodes?
• Are Y RNAs in RoRNPs involved in RNA degradation in vertebrates?
• What are the functions of non-canonical Y RNA-binding proteins and their associated RNPs?
• Are small RNA fragments derived from mammalian Y RNAs of functional significance and could they be used therapeutically?
• As a general outlook, why have so many different biological functions been compressed into such a small RNA molecule?
